# Next-Generation Sequencing for Diagnosis of Fatal Balamuthia Amoebic Encephalitis: A Case Report

**DOI:** 10.3390/diagnostics15202590

**Published:** 2025-10-14

**Authors:** Yuanyuan Feng, Huiyu Feng, Xuegao Yu, Jing Zhao, Hongyan Zhou, Jiaoxing Li, Peisong Chen, Li Feng

**Affiliations:** 1 Department of Clinical Laboratory, Children’s Hospital, Maternal and Child Health Hospital of Guangxi Zhuang Autonomous Region, Nanning 530002, China; fyyamy25@163.com; 2 Department of Neurology, Guangdong Provincial Key Laboratory of Diagnosis and Treatment of Major Neurological Diseases, National Key Clinical Department and Key Discipline of Neurology, First Affiliated Hospital, Sun Yat-sen University, Guangzhou 510080, China; fenghuiy@mail.sysu.edu.cn (H.F.); zhouhy7@mail.sysu.edu.cn (H.Z.); li.jiao.xing@163.com (J.L.); 3 Department of Laboratory Medicine, First Affiliated Hospital, Sun Yat-sen University, Guangzhou 510080, China; test0011012@163.com; 4 Department of Radiology, The First Affiliated Hospital, Sun Yat-sen University, Guangzhou 510080, China; 17707469646@163.com

**Keywords:** *Balamuthia mandrillaris*, encephalitis, next-generation sequencing, early diagnosis

## Abstract

**Background:** *Balamuthia mandrillaris* is a free-living amoebic parasite that primarily causes rare opportunistic infections in immunocompromised hosts. Balamuthia amoebic encephalitis (BAE) is a rare yet severe parasitic infection affecting the central nervous system. It has an extremely low incidence in China but can have a mortality rate as high as 98%. The clinical manifestations of amebic infections are similar to those of bacterial and tuberculous meningitis, lacking specificity, which makes accurate diagnosis challenging in the clinical setting. **Case Presentation:** A 61-year-old immunocompetent woman experienced worsening headache and a moderate fever over the course of five days, initially treated as a common cold. On 25 February 2025, she exhibited behavioral abnormalities, dysphagia, and a high fever of 40.2 °C, which progressed to a coma. On 26 February, her cranial CT scan revealed multifocal hemorrhagic lesions in the right frontotemporoparietal lobes. The MRI revealed similar lesions with slight enhancement and herniation. She underwent an emergency decompressive craniectomy, yet her condition continued to deteriorate following the surgery. On 27 February, serum targeted next-generation sequencing (tNGS) detected *B. mandrillaris*. Additionally, metagenomic NGS (mNGS) of the cerebrospinal fluid (CSF) sample confirmed the presence on 28 February. Finally, *B. mandrillaris* was identified through a brain tissue biopsy on 3 March. However, due to the delayed diagnosis and lack of effective drugs, her condition rapidly deteriorated and became irreversible. Her family ultimately chose to withdraw treatment. **Conclusions:** This study highlights the application of NGS for early diagnosis of patients with severe CNS infection. Both tNGS and mNGS can be considered for the rapid detection of rare or novel pathogens and for facilitating diagnosis.

## 1. Introduction

*Balamuthia mandrillaris* is a free-living ameba that can survive independently in nature without a host, obtaining nutrients through predation and the decomposition of organic matter. The U.S. Centers for Disease Control and Prevention (CDC) first discovered *Balamuthia* ameba in the brain of a baboon that died from meningitis in 1986 and identified it as *B*. *mandrillaris* in 1993 [1]. Individuals of any age are vulnerable to *B. mandrillaris*. Infection with *B*. *mandrillaris* can lead to a variety of clinical manifestations in human, typically skin lesions as the initial symptom. This may be followed by nasopharyngeal or sinus infections, and even Balamuthia amoebic encephalitis (BAE) in severe cases. Previously, over 300 BAE cases have been reported globally, with approximately a few dozen reported in China [2]. Case numbers have surged in parallel with the expanding scope of human impact on the environment. BAE generally exhibits a subacute or chronic progression, characterized by an insidious but progressive onset [3]. The duration of the latency phase remains unclear, varying from several weeks to months after exposure. The primary clinical manifestations include headache, fever, nausea, drowsiness, psychiatric disturbances, seizures, epilepsy, hemiparesis, hallucinations, ataxia, and bradykinesia. Ultimately, the majority of BAE cases result in death.

The nonspecific clinical symptoms, combined with the rapid progression of the disease, significantly complicate the process of achieving a timely and accurate diagnosis. Furthermore, BAE presents significant therapeutic challenges due to the lack of targeted and effective treatment options, which contributes to a notably high mortality rate. This paper aims to enhance healthcare professionals’ understanding of amebic encephalitis and to underscore the clinical significance of next-generation sequencing (NGS) in the early diagnosis of unexplained meningitis. It also emphasizes that in some critically ill patients with central nervous system infections, high-sensitivity targeted next-generation sequencing (tNGS) or metagenomic next-generation sequencing (mNGS) can assist in the timely identification of rare pathogens.

## 2. Case Presentation

The patient, a 61-year-old female, was admitted to our hospital on 25 February 2025, presenting with a primary complaint of fever and headache for eight days, behavioral abnormalities for two days, and altered consciousness for one day. Initially, the patient presented with an unbearable headache and a moderate fever below 38.5 °C. After being treated with ibuprofen and hurb capsules at the local outpatient clinic, these symptoms showed no significant relief. On 23 February, she exhibited signs of a mental disorder, including defecating in the bedroom, and was taken to the hospital in town. Her cranial magnetic resonance imaging (MRI) scan suggested an infarction in the right frontotemporal lobe of the brain. Consequently, she was diagnosed with an ischemic stroke and treated with clopidogrel and indobufen for antiplatelet therapy, atorvastatin for lipid reduction, and steroids (methylprednisolone and dexamethasone) to control fever. However, the patient’s symptoms continued to deteriorate. On 24 February, the patient developed a fever of 40.2 °C and became comatose. Subsequently, she was admitted to our hospital on 25 February.

**Figure 1:**
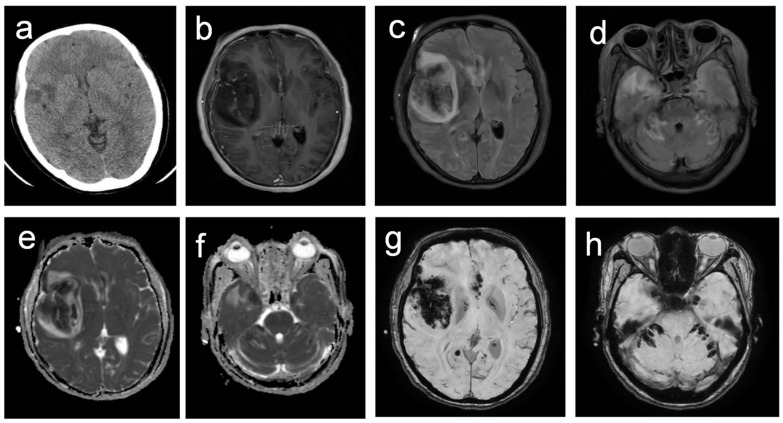
Neuroimaging findings in *B. mandrillaris* amebic encephalitis demonstrating multifocal hemorrhagic lesions. (**a**) Non-contrast CT reveals small, irregular, hypodense lesions in the right frontal and temporal lobes, indicating infarction. (**b**) T1-enhanced image reveals slightly enhanced lesions with adjacent meningeal enhancement. (**c**,**d**) T2-FLAIR sequences reveal scattered bilateral lesions in the frontal and temporal lobes, cerebellum, and brainstem, characterized by central hypointensity surrounded by hyperintensity. (**e**,**f**) Diffusion-weighted imaging confirms restricted diffusion in all lesions.; (**g**,**h**) Susceptibility-weighted imaging reveals multiple irregular hypointense foci, consistent with hemorrhagic components.

Upon admission, the patient was diagnosed with a central nervous system (CNS) infection accompanied by a secondary ischemic stroke. Empirical antiviral and antibacterial therapies were initiated, including a meropenem 2 g IV drip every 8 h, acyclovir 0.375 g IV drip every 8 h, and vancomycin 0.5 g every 6 h. A lumbar puncture was performed immediately, and the initial pressure was found to exceed 330 mmH_2_O. The results of the laboratory tests are listed in Table 1. To control the increasing intracranial pressure, mannitol 125 mL was administered every 8 h and albumin 50 mL twice daily. The cranial computed tomography (CT) conducted on 26 February revealed a hemorrhage in the right frontal, parietal, and temporal lobes, accompanied by adjacent brain edema and compression of the right lateral ventricle (Figure 1a). On the same day, the cranial MRI confirmed a mass-like abnormal signal in the right frontal, parietal, and temporal lobes, accompanied by right entorhinal herniation (Figure 1b–h). The patient underwent an emergent decompressive craniectomy immediately, followed by a multidisciplinary consultation. Given the rapid progression, secondary involvement of cranial vessels, imaging features, and the patient’s farming background, rhino-orbito-cerebral mucormycosis (ROCM) was strongly suspected. Voriconazole was then added.

**Figure 2:**
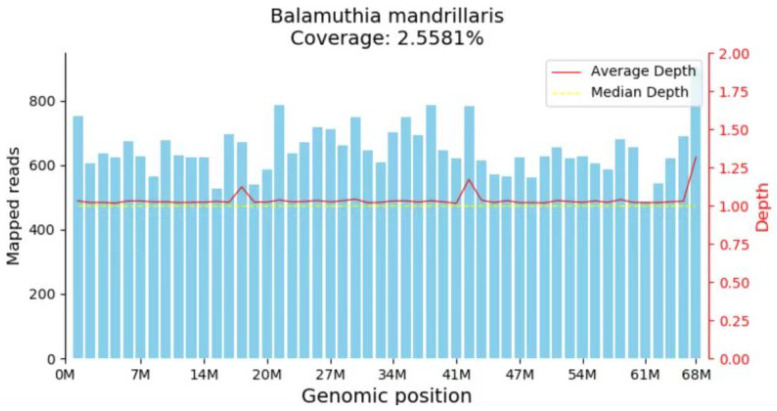
mNGS indicated the mapped read number was distributed across the genomic region of *B. mandrillaris*.

In addition to the routine negative tests for pathogens, the serum sample submitted for tNGS on 25 February tested positive for *Balamuthia mandrillaris* on 27 February (DNA sequences: 4, coverage: 1.03%, relative abundance: 100%), as well as for human herpesvirus 5 (DNA sequences: 110, coverage: <0.01%, relative abundance: 42.33%) and human herpesvirus 4 (DNA sequences: 90, coverage: <0.01%, relative abundance: 51.74%) (Table 2). Similarly, metagenomic next-generation sequencing (mNGS) of the cerebrospinal fluid confirmed the presence of *B. mandrillaris* on 28 February (DNA sequences: 49,517, coverage: 2.6%, relative abundance: 100%), with no other pathogens detected (Table 3). The genomic coverage map is depicted in Figure 2.

**Figure 3:**
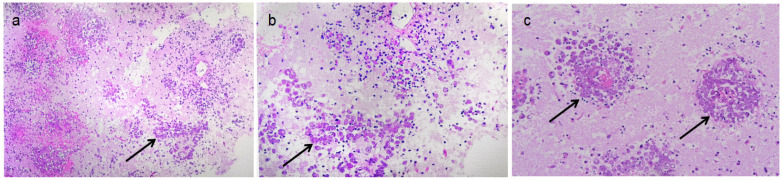
Amebic trophozoites verified by biopsy. (**a**,**b**) Focal neutrophil infiltration was observed alongside numerous oval cell clusters arranged in a nest-like distribution (arrow showed); (**c**) Biopsy showed Amoebic trophozoites (arrow showed).

Additionally, a small piece of brain tissue from the surgery was sent for pathological examination. The immunohistochemical results were as follows: CK (−), CD68 (−), NF (no axonal structures observed in the proximal tissue). The immunohistochemistry results excluded common epithelial-derived tumors and macrophage-associated diseases, whereas the histological analysis revealed severe damage to the brain. Special staining revealed: Gomori’s methenamine silver (GMS) (staining for fungi yielded a result of (+/−), PAS staining was positive, and hematoxylin-eosin (staining showed blue staining. The results of the three special stains, particularly the positive staining of amoebic trophozoites with hematoxylin, definitively confirmed the diagnosis of amoebic encephalitis on 3 March (Figure 3a–c).

**Figure 4:**
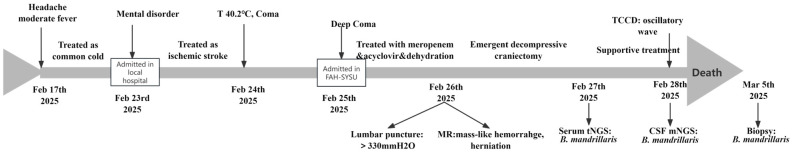
The main events along with disease proceeding.

However, despite surgery and dehydration treatment, the patient’s intracranial pressure continued to rise. On 28 February, the patient was in a deep coma, and her transcranial coloured Doppler (TCCD) showed oscillatory waves. Given her critical condition and the absence of specific drugs for amoebic encephalitis, her family made the difficult decision to withdraw treatment, and the patient passed away on the same day. The entire disease progression and intervention process are detailed in Figure 4.

This study was approved by the Ethics Committee for Clinical Research and Animal Trials of the First Affiliated Hospital of Sun Yat-sen University (2023-331). Written informed consent has been obtained from the patient to publish this paper.

## 3. Discussion

As mentioned, *B. mandrillaris* was first identified in 1986 by the CDC of USA and nominated in 1993 [1]. In 2003, Schuster et al. [4] first successfully isolated Balamuthia from soil and identified it using immunofluorescence methods. Studies have shown that the genome of *B. mandrillaris* is particularly similar to that of Acanthamoeba. Booton et al. [5] demonstrated high similarity between the genomes of Balamuthia and Acanthamoeba through 18S RNA gene sequencing, while mitochondrial 16S rRNA gene sequencing showed only a 1.8% difference [6]. Similarly to Acanthamoeba, *B mandrillaris* is widely distributed in soil and water [4,7,8]. Consequently, Balamuthia found in freshwater, soil, and dust serves as a primary source of infection. The amoeba can infect through damaged skin, nasal mucosa [9], or the cornea. Sometimes, it may also be inhaled through the respiratory system, subsequently invading the body via the olfactory nerve. Additionally, it can infect the central nervous system through the digestive system after initial infection via the bloodstream, resulting in granulomatous amoebic encephalitis (GAE) [10]. The reports from BAE are coming from all over the world, and the case number has been increasing in recent years. It is indicated that incidence rates are higher in the tropical and subtropical regions of North America and Latin America [11,12]. However, cases have also been reported in China [13], Iran [14], and Japan [11]. The unclear relationship with environmental factors, genetic predisposition, limited medical conditions, and socio-economic factors remains to be fully understood [15]. The susceptible populations include those with organ transplants, cancer, or chronic underlying diseases, as well as those in areas with poor sanitary conditions [3,16]. Cases have also been reported among individuals with normal immunity and children [17,18,19]. It is indicated that macrophages have no inhibitory effect on Balamuthia in vitro, suggesting that both immunocompetent and immunocompromised individuals can be infected by *Balamuthia* [20]. in this instance, the patient was immunocompetent, and her route of infection remains unclear. The poor hygiene condition with a chicken feces environment may contribute to her infection.

Clinical manifestations after infection with *B. mandrillaris* vary a lot, usually presenting with skin lesions and pulmonary symptoms, which can last for months or even years. The BAE is a rare but life-threatening disease, with a short incubation period as about 5 days and non-specific clinical symptoms. It usually manifests with insidious symptoms resembling nasopharyngitis, followed by fever, headache, nausea, vomiting, and altered consciousness [21]. The poor outcome of BAE is usually associated with the triad of high virulence of *B. mandrillaris*, immune evasion [2], diagnostic delay, and fulminant CNS inflammation and tissue necrosis following blood–brain barrier breaching.

Routine laboratory tests were less indicative in identifying *B. mandrillaris*. The cellular and biochemical alterations in both serum and cerebrospinal fluid (CSF) are nonspecific. In this instance, all observed changes in serum and CSF, including electrolytes, glucose levels, and plasma osmolality, can be attributed to secondary dysfunction resulting from severe brain damage and intracranial hypertension. A routine examination of the CSF revealed a slight increase in the white blood cell count to 29 × 10^6^/L, predominantly composed of neutrophils at 70%, along with elevated protein levels. These changes were consistent with those of previous reports and were nonspecific, being observable in various intracranial infections [22]. Likewise, the imaging characteristics of BAE may present as space-occupying lesions, cerebral edema, and focal necrosis [18], potentially resembling abscesses or ring-enhancing lesions [23]. When vessels are involved, as in our case, patients may present with images of intracranial hemorrhage and stroke, leading to misdiagnosis as cerebrovascular diseases or ROCM, which is also usually invaded into cerebral vessels and resulted in secondary stroke or hemorrhage [24].

Therefore, diagnosing BAE poses a significant challenge for clinicians, necessitating a highly effective method. Traditionally, the laboratory diagnostic methods for BAE encompass immunohistochemistry, polymerase chain reaction (PCR), specific serum antibodies, and tissue biopsy. Additionally, staining microscopy offers opportunities to identify unique trophozoites or cysts in skin, brain tissue, or cerebrospinal fluid. Generally, PCR offers high sensitivity and rapid turnaround for detecting pathogens, but it requires pre-specified targets. Serology offers evidence of an immune response, yet it may have a window period and exhibit cross-reactivity. Immunohistochemistry(IHC) offers in situ pathogen detection and morphological correlation, but it relies on high-quality tissue and specific antibodies. More importantly, all the methods mentioned require clinicians to consider BAE, which is challenging due to the rarity of the disease. Missing the very narrow window for diagnosis, BAE can lead to rapid progression and severe neurological damage, and even death. In addition, most of these methods are unavailable in clinical laboratories due to the absence of commercial reagents, which hinders timely diagnosis.

NGS technology takes the advantages of unbiased identification of all the pathogens in a sample without specific targets. In this instance, NGS revealed the presence of BAE within two days, a possibility that had been beyond the consideration of clinicians. The result was eventually confirmed by biopsy, highlighting the potential benefit of NGS in rapid diagnosing rare pathogens, particularly in cases of severe intracranial infections. Technologically, mNGS is a high-throughput sequencing technique that enables unbiased detection of thousands to tens of thousands of microorganisms—including bacteria, fungi, viruses, and parasites—by sequencing both DNA and RNA extracted from clinical samples. Its unbiased detection particularly facilitates the identification of rare, novel, or unusual pathogens. Moreover, it has the capability for comprehensive functional analysis of microbial communities, extending beyond taxonomic classification [25]. However, the sensitivity of mNGS is sometimes compromised by high levels of host nucleic acids, making the increase in pathogen-derived reads a critical factor in ensuring detection accuracy.

In contrast, tNGS offers targeted detection with minimal influence from host nucleic acids—methods based on multiplex or super-multiplex PCR are generally unaffected, while probe capture-based approaches show less susceptibility than mNGS. It utilizes pre-sequencing enrichment or amplification of nucleic acids from specific pathogens, followed by high-throughput sequencing and bioinformatic analysis. This method significantly increases both the quantity and proportion of pathogen sequences, thereby enhancing detection sensitivity. Compared to conventional diagnostic methods, tNGS is also capable of simultaneously detecting hundreds of pathogens, offering broader coverage and higher sensitivity. By focusing on a predefined set of targets, tNGS also reduces the volume of sequencing data and associated costs, thereby alleviating the economic burden on healthcare systems. However, advantages of higher sensitivity, lower cost, and faster turnaround times are associated with its predefined panel of pathogens, thereby organisms not covered in its bank cannot be identified. Nevertheless, the bank of tNGS can cover most identified pathogens, as shown by our case, both tNGS and mNGS successfully identified the *B. mandrillaris*, making it an economic-effective choice for diagnosis. In total, these two methodologies are highly effective and complementary to each other. Clinicians can make an optimal choice based on the severity of the disease and the patient’s economic background, or they can consider these factors sequentially.

*Herpesviruses* IV and V are highly prevalent in the general population and can establish lifelong latent infections. In most cases, these infections are asymptomatic; severe manifestations typically occur only in infants with congenital infections or in individuals with compromised immune systems. In this instance, the detection of *herpesviruses* IV and V in the serum likely indicates previous exposure. The result verified the sensitivity of NGS from another aspect.

The extremely high mortality of BAE at 98% is mainly attributed to delayed diagnosis coupled with a lack of effective treatments. The CDC of USA published a case report in 2017 recommending the combined use of miltefosine, azithromycin, fluconazole, flucytosine, sulfadiazine, and macrolide antibiotics for antiinfection management [1,26]. However, treatment efficacy remains suboptimal, primarily due to the presence of the blood–brain barrier and the thick-walled structure of amoebic cysts. To date, there have been only a limited number of BAE cases successfully treated. In 2014, Moriarty et al. [27] described a 4-year-old patient with BAE who experienced a favorable outcome after undergoing miltefosine therapy. More recently, in 2023, Spottiswoode et al. [28] reported on a 50-year-old BAE patient who responded well to a combination regimen consisting of nitazoxanide, miltefosine, azithromycin, albendazole, fluconazole, and low-dose fluorouracil. A review of the relevant literature revealed a serial of successful use of APL-1202 (nitroxoline tablets) in treating BAE [29]. All successful cases shared the common feature of timely diagnosis. Thus, the key for patients to survive BAE is to make the correct diagnosis promptly. Delayed diagnosis leads to widespread and severe damage to the central nervous system, and the prognosis is poor [29,30].

BAE remains a rare disease in China, with only a few dozen cases reported nationally. However, the incidence of BAE has been increasing in recent years, which may be associated with climate changes, including warming and expanding areas of human activities. However, considering BAE initially remains challenging for clinicians in non-endemic areas. Even when they do consider it, the lack of access to targeted identification methods, such as PCR, still compromises timely diagnosis. This case, even with an unfavorable outcome, highlights the advantage of NGS, whether it be tNGS or metagenomic NGS (mNGS), in identifying rare pathogens and facilitating treatment. The NGS technology may hold promise for future therapeutic breakthroughs [31,32,33].

## 4. Conclusions

BAE is a rare and challenging disease without specific symptoms, routine laboratory tests or images. In this instance, both high-sensitivity tNGS and mNGS successfully played a crucial role in identifying the amoebic pathogen. These findings highlight the utility of NGS in providing rapid and accurate pathogen detection for encephalitis of unknown pathogens. NGS technology may secure a critical time window for targeted intervention and potentially lower mortality attributed to delayed diagnosis. The early application of NGS in severe cases with suspected intracranial infection is recommended to improve clinical decision process and outcomes.

## Figures and Tables

**Table 1:** The results of the laboratory tests upon admission to FAH-SYSU.

Laboratory Examination	Items	25 February	26 February	27 February	Range
Complete blood count	White blood cell (×10^9^/L)	9.83	11.87 ↑	9.26	4.00–10.00
	Neutrophil,%	0.825 ↑	0.920 ↑	0.899 ↑	0.460–0.750
	CRP (mg/L)	4.81	30.96 ↑	47.57 ↑	0.00–10.00
Biochemistry	PCT (ng/L)	0.18 ↑	0.37 ↑	0.34 ↑	0.00–0.05
	Sodium (mmol/L)	130 ↓	151 ↑	170 ↑	135–145
	Potassium (mmol/L)	2.74 ↓	2.68 ↓	2.99 ↓	3.5–5.3
	Chloridion (mmol/L)	97	118 ↑	136 ↑	96–110
	Blood glucose (mmol/L)	8.0↑	18.2 ↑	17.3 ↑	2.9–6.0
	Serum osmolality (mOsm/L)	274.0 ↓	325.1 ↑	359.9 ↑	275–295
CSF biochemistry	Glucose (mmol/L)	-	-	4.2 ↑	2.3–3.9
	Chloride (mmol/L)	-	-	144 ↑	119–129
	Protein (mg/L)	-	-	12,114.5 ↑	120.0–600.0
CSF microscopic examination	White blood cell (×10^6^/L)	-	-	29 ↑	≤10
	Mononuclear cell,%	-	-	0.3	0–0.6
	Multinucleate cell,%	-	-	0.7 ↑	0–0.4
Cultures of the CSF	Culture of bacteria, fungi and tuberculosis bacilli	negative	negative	negative	negative
Tuberculosis antibody and tuberculosis smear examination	Tuberculosis antibody and tuberculosis smear examination	-	negative	-	negative
fungal glucan test (G test)	fungal glucan test (G test)	-	negative	-	negative
Mycological Triple Panel Test	Detection of Aspergillus, Cryptococcus and Pneumocystis nucleic acids	-	negative	negative	negative

CRP: C-reaction protein; PCT: procalcitonin; CSF: cerebrospinal fluid; ↑: increase; ↓: decrease.

**Table 2:** The micro-organisms were detected by tNGS (Venous blood).

Category	Pathogen	Reads	Coverage (%)	Relative Abundance (%)
Bacteria	-	-	-	-
Fungi	-	-	-	-
Virus	*Human Herpesvirus V*	110	<0.01	42.33
	*Human Herpesvirus IV*	90	<0.01	51.74
Parasite	*B.* *mandrillaris*	4	1.03	100
Human microbiota	-	-	-	-

**Table 3:** The micro-organisms were detected by mNGS (Cerebrospinal fluid).

Category	Pathogen	Reads	Coverage (%)	Relative Abundance (%)
Bacteria	-	-	-	-
Fungi	-	-	-	-
Virus	-	-	-	-
Parasite	*B.* *mandrillaris*	49,517	2.6	100
Human microbiota	-	-	-	-

## Data Availability

Data or detailed information can be obtained from the corresponding authors on request.
